# Comprehensive Transcriptome Analysis Expands lncRNA Functional Profiles in Breast Cancer

**DOI:** 10.3390/ijms25158456

**Published:** 2024-08-02

**Authors:** Wenyong Zhu, Hao Huang, Zixi Hu, Yu Gu, Rongxin Zhang, Huiling Shu, Hongjia Liu, Xiao Sun

**Affiliations:** State Key Laboratory of Digital Medical Engineering, School of Biological Science and Medical Engineering, Southeast University, Nanjing 211189, China

**Keywords:** long non-coding RNA, functional genome, molecular subtyping of breast cancer

## Abstract

Breast cancer is a heterogeneous disease that arises as a multi-stage process involving multiple cell types. Patients diagnosed with the same clinical stage and pathological classification may have different prognoses and therapeutic responses due to alterations in molecular genetics. As an essential marker for the molecular subtyping of breast cancer, long non-coding RNAs (lncRNAs) play a crucial role in gene expression regulation, cell differentiation, and the maintenance of genomic stability. Here, we developed a modular framework for lncRNA identification and applied it to a breast cancer cohort to identify novel lncRNAs not previously annotated. To investigate the potential biological function, regulatory mechanisms, and clinical relevance of the novel lncRNAs, we elucidated the genomic and chromatin features of these lncRNAs, along with the associated protein-coding genes and putative enhancers involved in the breast cancer regulatory networks. Furthermore, we uncovered that the expression patterns of novel and annotated lncRNAs identified in breast cancer were related to the hormone response in the PAM50 subtyping criterion, as well as the immune response and progression states of breast cancer across different immune cells and immune checkpoint genes. Collectively, the comprehensive identification and functional analysis of lncRNAs revealed that these lncRNAs play an essential role in breast cancer by altering gene expression and participating in the regulatory networks, contributing to a better insight into breast cancer heterogeneity and potential avenues for therapeutic intervention.

## 1. Introduction

Breast cancer is the most prevalent malignant tumor, with high morbidity and mortality rates, accounting for most cancer diagnoses in women. Despite the significant advances in breast cancer research over the past decade, the diagnosis and treatment of this malignant tumor remains a formidable challenge [1,2,3]. Breast cancer is a highly heterogeneous malignant tumor in terms of histological and molecular classification [2]. Clinically, breast cancer is commonly classified into different subtypes based on a variety of immunohistochemical markers, including estrogen receptor (ER), progesterone receptor (PR), human epidermal growth factor receptor (HER2), and tumor proliferation antigen (Ki-67), which are utilized to guide the diagnostic decisions and clinical treatment [4]. Nevertheless, molecular subtyping has been demonstrated to facilitate a more comprehensive classification based on gene expression profiling [5], to be more sensitive to the immune status, and to enhance the prediction of recurrence and distant metastasis [6], which is crucial for personalized treatment strategies and better patient outcomes. For example, PAM50 is a molecular subtyping criterion that is extensively applied to classify intrinsic subtypes of breast cancer based on the expression profiling of 50 genes and is intimately related to disease diagnosis, prognosis, and treatment response [7]. It has been well-studied that even if the clinical stage and pathologic classification of breast cancer are identical, alterations in molecular genetics can contribute to divergent prognoses and therapeutic responses [8]. Therefore, further investigation into the molecular and genetic regulatory mechanisms will facilitate novel insights into carcinogenesis, as well as promote the diagnosis and prognosis of breast cancer.

As essential signatures for molecular subtyping of breast cancer, long non-coding RNAs (lncRNAs) play crucial roles in the regulation of gene expression, cellular differentiation, and the maintenance of genomic stability [9], and their aberrant expressions are intricately associated with the pathogenesis of breast cancer [10]. Furthermore, the complex interactions between lncRNAs and protein-coding genes, involved in crucial signaling pathways, can unravel the molecular mechanisms of breast cancer heterogeneity [11]. Despite a widely acknowledged potential for several lncRNAs as a category of disease biomarkers [12,13], a comprehensive and systematic landscape for the biological functions and regulatory mechanisms of lncRNA have yet to be sufficiently characterized. Moreover, except for the annotated lncRNAs, a vast quantity of novel lncRNAs remain to be identified, and the regulatory differences associated with lncRNAs in various molecular subtypes remain to be further investigated.

In this study, we developed a novel framework for identifying unannotated lncRNAs using RNA-seq data and applied it to a breast cancer cohort (SEU-BRCA) [14] to explore the potential biological function and regulatory mechanisms of lncRNAs. Subsequently, the genomic and chromatin features of the unannotated lncRNAs, defined as novel lncRNAs, were elucidated, along with the associations between protein-coding genes and putative enhancers that physically overlap with these lncRNAs. Furthermore, we unraveled the differences in the biological functions and molecular mechanisms of the novel and annotated lncRNAs in PAM50-based breast cancer subtypes. Ultimately, we performed unsupervised clustering based on the expression patterns of the identified lncRNAs to the effects of different clusters on hormone receptor characterization and immune activation. Taken together, the comprehensive identification and functional analysis of lncRNAs in breast cancer will facilitate the perception of breast cancer heterogeneity, promote breast cancer diagnosis and prognosis, and potentially provide new avenues for therapeutic intervention.

## 2. Results

### 2.1. A Framework for the Identification of Novel lncRNAs in the SEU-BRCA Dataset

**Figure 1:**
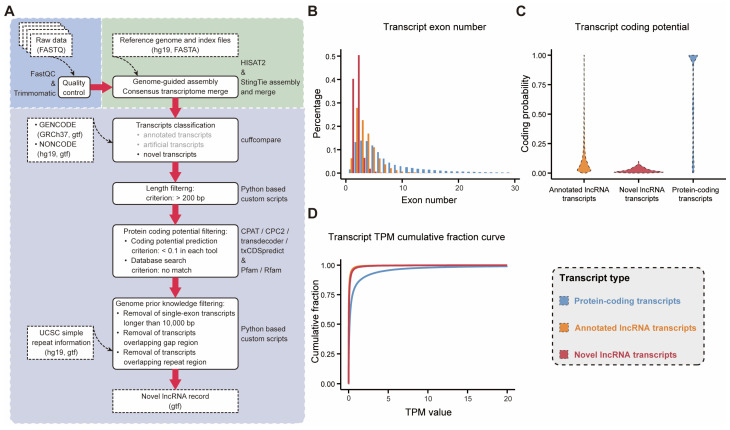
Novel lncRNA identification and comparison of multiple transcript characterization. (**A**) Workflow of RNA-seq-based procedures for novel lncRNA identification. Raw data preprocessing, transcriptome reconstruction, and transcript filtering are shown in blue, green, and grey as backgrounds, respectively. Solid boxes indicate the processing that was performed, while dashed boxes indicate the files that were used or generated; Comparison of (**B**) the number of transcript exons, (**C**) coding potential, and (**D**) transcript expression, between protein-coding transcripts, annotated lncRNA transcripts and novel lncRNA transcripts. TPM stands for transcript per million.

RNA sequencing (RNA-seq), as an indispensable tool for transcriptome-wide analysis of differential gene expression and differential splicing of mRNAs, has shaped almost every aspect of our understanding of genomic function [15]. Currently, a lot of RNA-seq-based methods have been proposed for the identification of lncRNAs [16,17,18,19], yet the computational methods employed for lncRNA identification remain diverse and insights into the underlying functions of the identified lncRNAs are lacking. We synthesized prior knowledge on the effectiveness of software tool applications [20,21] to optimize a lncRNA identification framework and applied it to the SEU-BRCA dataset, containing 199 tumor tissues from breast cancer patients (Figure 1A). The workflow includes three modules: raw data preprocessing, transcriptome reconstruction, and candidate lncRNA identification. In particular, the process of candidate lncRNA identification involves four steps: (a) remove annotated and pseudo-transcripts; (b) retain transcripts longer than 200 bp; (c) require transcripts with no protein-coding potential; and (d) extract novel lncRNA transcripts based on prior knowledge of the annotated lncRNA.

Our study identified 9831 novel lncRNA transcripts corresponding to 9108 novel lncRNA genes, which were not reported in the NONCODE database and suggested that some of these novel lncRNA genes could correspond to multiple variably spliced formats of novel transcripts. To verify the credibility of the identification framework, we characterized the novel lncRNAs in comparison with the already annotated lncRNAs and protein-coding genes. We extracted the protein-coding transcripts and lncRNA transcripts, respectively, that had been annotated in GENCODE, and performed comparisons with the novel lncRNAs in the following three aspects: transcript exon number, transcript coding potential, and transcript expression. In terms of transcript exon number comparison, the annotated lncRNAs and the novel lncRNAs had fewer exons than the protein-coding transcripts (Figure 1B). Meanwhile, both the annotated lncRNAs and the identified novel lncRNAs were observed to harbor weaker protein-coding capabilities, in contrast to protein-coding transcripts, whose protein-coding capacity values were distributed centrally in the range of 0.9 to 1 (Figure 1C). Regarding transcript expression, both annotated and novel lncRNA expression were lower compared to protein-coding transcripts (Figure 1D). In addition, a pipeline to identify lncRNAs based on feature relationship, designated as LGC [22], was utilized. It was demonstrated that the novel lncRNAs we identified were substantially validated, accounting for 99.40% (9772/9831) of the total. Overall, the novel lncRNAs identified in SEU-BRCA were consistently identical to the annotated lncRNAs in the three features and different from the protein-coding genes, which supported the credibility of our customized workflow to identify novel lncRNAs in a breast cancer cohort.

### 2.2. Novel Intronic lncRNAs Involved in Breast-Cancer-Specific Biological Processes

**Figure 2:**
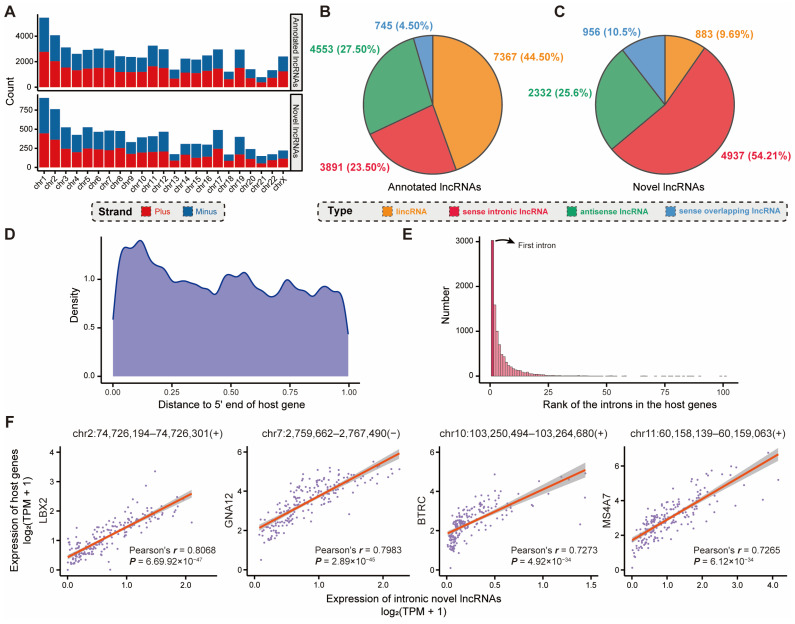
Genome-wide characterization of novel lncRNAs in breast cancer. (**A**) Distribution of annotated lncRNA genes from GENCODE and novel lncRNA genes identified from SEU-BRCA on chromosomes; Classification of (**B**) annotated lncRNA genes, and (**C**) novel lncRNA genes; (**D**) Normalized distance from the middle points of intronic lncRNAs to the 5′ ends of the corresponding host genes; (**E**) Rank of the introns in the host genes where the novel intronic lncRNAs were located; (**F**) the expression correlation analysis between the four intronic novel lncRNAs and the host genes, where each point represents a sample and the solid orange lines are the fitted lines.

lncRNAs are implicated in the intricate spatial and temporal regulation of gene expression, relying on sequence-encoded localization signals [23]. Our study statistically characterized the distribution of the annotated and novel lncRNA genes on both the plus and minus strands of each chromosome (Figure 2A). It was evident that the novel lncRNA genes identified in SEU-BRCA maintained a high degree of consistency with the annotated lncRNA genes in both chromosome and chromosomal strand distribution. In addition, the majority of annotated lncRNAs retrieved from GENCODE were situated in the intergenic regions, accounting for 44.50% of the total (Figure 2B), while the novel lncRNAs were predominantly located in the intronic regions, accounting for 54.21% of the overall (Figure 2C, $p<1\times 10^{-16}$, Chi-square test). Meanwhile, intronic lncRNAs also exhibited a tendency to bias toward the 5′ end of the host gene body (Figure 2D,E and Figure S1A,B), which could facilitate alterations in host gene expression. In view of the fact that the genomic regions where protein-coding genes reside are able to transcribe functional lncRNAs [24], these novel intronic lncRNAs potentially correlate with expression patterns specific to breast cancer.

To gain further insight into the underlying mechanism governing the distribution pattern of the novel intronic lncRNAs, we conducted functional annotation analysis on the protein-coding genes detected through intronic lncRNA localization, named host genes. Then, the host genes were discovered to be involved in several relevant pathways that have been widely discussed in cancer research (Supplementary Figure S1C,D). Particularly, several studies have spotlighted the essential roles that some of these pathways play in breast cancer [25,26], such as the Wnt signaling pathway, RAS protein signal transduction, ErbB signaling pathway, and proteoglycans in cancer (see Discussion for more details). For example, the Wnt signaling pathway participates in the proliferation and metastasis of mammary tumors and acts in the regulation of the immune microenvironment, stem cell maintenance, therapeutic resistance, and maintenance of cell morphology in breast cancer [27]. It was also enriched with epigenetic pathways, including covalent chromatin modification, histone modification, and chromatin remodeling, which are crucial approaches for lncRNAs to exert their functions [28]. Moreover, expression correlation analysis between the above intronic lncRNAs and the host genes yielded four novel lncRNAs that were significantly and strongly correlated with cancer-related genes (Pearson correlation coefficient r>0.7 and p<0.01, Figure 2F). For example, it has been demonstrated that one of these lncRNAs (chr2:74,726,194–74,726,301), which is most relevant to host gene expression, could upregulate *LBX2* expression via enhanced mRNA stability, and the overexpression of *LBX2* correlates with tumor progression and poor prognosis in cancer [29,30]. In addition, the other three host genes (*GNA12*, *BTRC*, and *MS4A7*) correlated to lncRNA expression have been demonstrated to be associated with cancer progression [31,32,33], and specifically, the enhancement of *BTRC* expression could facilitate M2 macrophage polarization in breast cancer [33]. Additionally, we utilized MEME Suite to predict enriched miRNA motifs within these novel lncRNAs and discovered that all novel lncRNAs possessed at least one miRNA motif. Among enriched miRNA motifs, hsa-miR-8089 (Bonferroni adjusted $p=2.95\times 10^{-224}$, Fisher’s exact test) was enriched by the maximum number (4843, 49.26%) of the novel lncRNAs, which was demonstrated to be associated with the appearance of new distant metastases in breast cancer [34]. Overall, the observed results suggested that the novel lncRNAs were predominantly located in intronic regions and were involved in breast-cancer-related biological processes, suggesting that these novel lncRNAs could have an essential role in various aspects of breast cancer progression.

### 2.3. Novel lncRNAs Associated with Putative Enhancers Were Involved in Breast Cancer Regulatory Network

**Figure 3:**
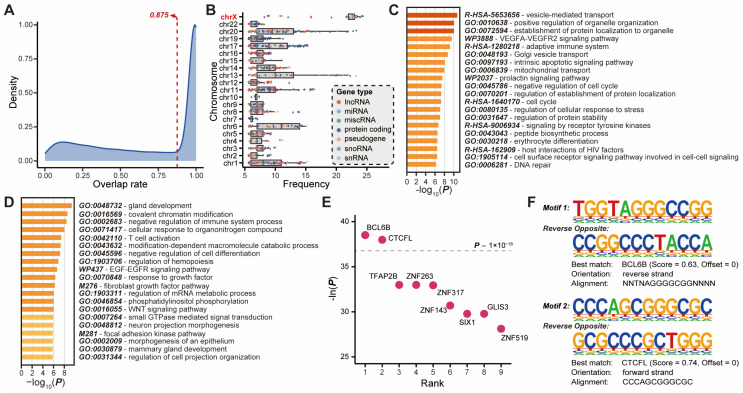
Alignment of known regulatory elements related to novel lncRNAs. (**A**) Overlap rate distribution of breast cancer-specific enhancers overlapping with novel lncRNAs; (**B**) Statistics of high-frequency target genes for novel lncRNAs with respect to chromosome; (**C**) Functional enrichment of high-frequency target genes for novel lncRNAs; (**D**) Functional enrichment and (**E**) motif enrichment of target genes correlated with novel lncRNA expression levels; (**F**) the two most significant motifs enriched by novel lncRNA genes with best matching transcription factors.

Based on the association with DNA regulatory elements of known function, a subset of lncRNAs can be classified as enhancer lncRNAs (elncRNAs) [31]. Accumulating evidence suggested that elncRNAs play a critical role in binding to transcription factors and recruiting them to cognate enhancers, and are involved in various tumor biological processes, including cell proliferation, apoptosis, invasion, metastasis, and angiogenesis, as well as interacting with enhancers to regulate genes specific to cell identity [35,36]. To ascertain the potential functions of the novel lncRNAs with breast-cancer-specific enhancer features, we calculated the distribution of the overlap rate between novel lncRNAs and putative enhancers and selected the overlap rate where most of the overlaps were concentrated (Figure 3A), which contained 43.35% (3948) of the identified novel lncRNAs.

Evidence suggests that the transcripts of enhancers can influence the chromatin state and promote enhancer–promoter looping, thereby aiding in the transcriptional activation of target genes [37]. A total of 1290 genes targeted by novel-lncRNA-associated enhancers were obtained from EnhancerAtlas 2.0 (Methods). Subsequently, the chromosomal origin statistics for these genes revealed a significantly higher frequency of target genes originating from the X chromosome compared to genes on other chromosomes (Figure 3B). For example, the *FLNA* gene, located on the X chromosome, is the target of the maximum quantity of novel lncRNAs. It was found that *FLNA* was overexpressed in breast cancer cells compared to normal breast tissue and the absence of *FLNA* could impede the formation of BRCA1 foci following DNA damage to promote breast cancer progression [38]. Furthermore, all target genes were subjected to functional enrichment analysis and clustered into pathways such as vesicle-mediated transport, VEGFA-VEGFR2 signaling pathway, intrinsic apoptotic signaling pathway, negative regulation of cell cycle, and so on (Figure 3C), which are associated with proliferation and metastasis of breast cancer.

Due to the inherent limitations of disparate datasets and the heterogeneity of breast cancers, the interaction pairs available in other datasets may inadvertently impede a comprehensive interpretation of all the information contained within the SEU-BRCA. Consequently, we calculated the expression correlation between each novel lncRNA overlaid with putative enhancers and the candidate target genes, resulting in 2631 interaction pairs comprising 979 novel lncRNAs and 2042 target genes (Methods). The functional enrichment analysis uncovered that these target genes are strongly relevant to the immune response such as negative regulation of immune system process and T cell activation (Figure 3D). Moreover, it was observed that these novel lncRNA genes, which overlapped with putative enhancers associated with breast cancer, exhibited a significant enrichment of nine transcription factor binding motifs (Figure 3E). The two most significant motifs exhibited the highest similarity to transcription factors BCL6B and CTCFL, respectively ($p<1\times 10^{-16}$, Figure 3F). Simultaneously, BCL6B has been identified as a potential driver of metastatic progression in breast cancer [39]. Additionally, CTCFL has the capacity to compete with CTCF for a subset of binding sites in cancer cells, thereby influencing chromosome architecture and gene regulation [40,41]. Aberrant CTCFL not only interferes with the functions of CTCF but also alters gene expression programs by acting as a transcriptional activator in oncogenesis [42].

### 2.4. Subtype-Specific lncRNAs Correlate with Dysregulated Pathways and Hormonal Characteristics

**Figure 4:**
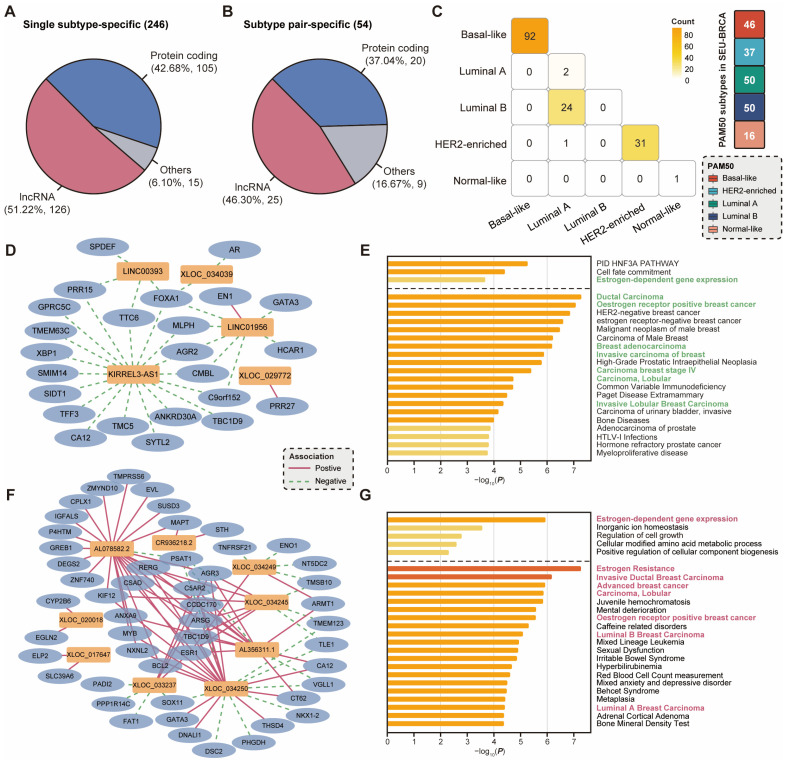
Subtype-specific expression patterns of lncRNAs in breast cancer. Types of (**A**) subtype-specific and (**B**) subtype-pair-specific expressed genes in breast cancer; (**C**) Heatmap of the number of co-expressed lncRNAs across five subtypes; (**D**) Co-expression network of specifically expressed lncRNAs and protein-coding genes in Basal-like; (**E**) Function annotation and disease enrichment of protein-coding genes negatively associated with specifically expressed lncRNAs in Basal-like; (**F**) Co-expression network of specific expressed lncRNA and protein-coding genes in subtype pairs of Luminal A and Luminal B; (**G**) Function annotation and disease enrichment of protein-coding genes positively associated with specifically expressed lncRNAs in subtype pair Luminal A and Luminal B.

The molecular expression profiles of breast cancers can be classified into multiple intrinsic subtypes, which have essential implications for clinical decisions on risk prediction and hormonal therapy for breast cancer. However, a few analogous characteristics persist among the molecular subtypes based on PAM50, including Luminal A, Luminal B, HER2-enriched, Basal-like, and Normal-like [43]. Meanwhile, although each subtype exhibits a distinct gene expression profile, there may still be similarities in the expression patterns of lncRNAs across different subtypes [44]. Therefore, to investigate the pathogenesis of different molecular subtypes of breast cancer, we utilized single subtype-specific or subtype-pair-specific (such as Luminal A and Luminal B, which are hormone receptor-positive) lncRNAs, which contained both novel and annotated lncRNAs. In our study, a total of 126 subtype-specific and 25 subtype-pair-specific lncRNAs were identified in SEU-BRCA (Figure 4A,B, Methods). Due to the uneven distribution of samples across PAM50 subtypes in the SEU-BRCA dataset, these specific lncRNAs were concentrated in a few subtypes or subtype pairs (Figure 4C). In particular, 92 subtype-specific lncRNAs were identified in Basal-like, 31 subtype-specific lncRNAs were detected in HER2-enriched, and 24 subtype-pair-specific lncRNAs were discovered in the Luminal A and Luminal B subtype pair (Figure 4C). Additionally, a comparison of subtype-specific protein-coding genes and lncRNA genes on primary breast tumor tissues from TCGA-BRCA and SEU-BRCA revealed that the trends in the quantitative distribution of specifically expressed genes in subtypes and subtype pairs were broadly consistent (Supplementary Figure S2), thereby corroborating that the amount of subtype-specific and subtype-pair-specific lncRNAs was reasonable. 

In order to gain further insight into the potential functions of each subtype-specific or subtype-pair-specific lncRNA, we constructed co-expression networks based on the correlation between these specifically expressed lncRNAs and associated protein-coding genes. The insufficient number of samples categorized as HER2-enriched in SEU-BRCA prevented the construction of the co-expression network for specifically expressed lncRNAs and protein-coding genes. Therefore, we focused on specifically expressed lncRNAs in subtype Basal-like and subtype pair Luminal A and Luminal B. In particular, the co-expression network, comprising specifically expressed lncRNAs and protein-coding genes in Basal-like, was primarily connected by negative correlation edges (Figure 4D), and these 22 protein-coding genes were involved in the pathways distinct from the characteristic of Basal-like. For example, the cell fate commitment pathway, which is associated with luminal and basal cell type switching, and the estrogen-dependent gene expression pathway, which is associated with estrogen levels (Figure 4E). Moreover, despite being significantly associated with breast cancer, these protein-coding genes were divergent from the immunohistochemical markers of Basal-like using the information of genes and variants associated with human diseases from DisGeNET (Figure 4E). This indicated that the specifically expressed lncRNAs in Basal-like could be associated with the deregulation of the breast cancer biological network and play an inhibitory role in the generation of the ER-positive breast cancer tumor cell phenotypes. Furthermore, the co-expression network of the subtype pair Luminal A and Luminal B comprises 35 protein-coding genes, which were connected by positive correlation edges to specifically expressed lncRNAs (Figure 4F). These protein-coding genes were also involved in the estrogen-dependent gene expression pathway and hormone receptor-positive (Figure 4G). It was plausible that the specifically expressed lncRNAs in the subtype pair Luminal A and Luminal B could be involved in the phenotype generation of the hormone-positive breast cancer tumor cells and play a significant role in hormone therapy resistance.

Taken together, the subtype-specific lncRNAs in Basal-like and subtype pair Luminal A and Luminal B were associated with hormonal characteristics, correctly reflected different immunohistochemical indicators across subtypes or subtype pairs, and correlated with perturbations in breast cancer biological networks.

### 2.5. Expression Patterns of lncRNAs Associated with Hormonal Characteristics and Immune Activation

**Figure 5:**
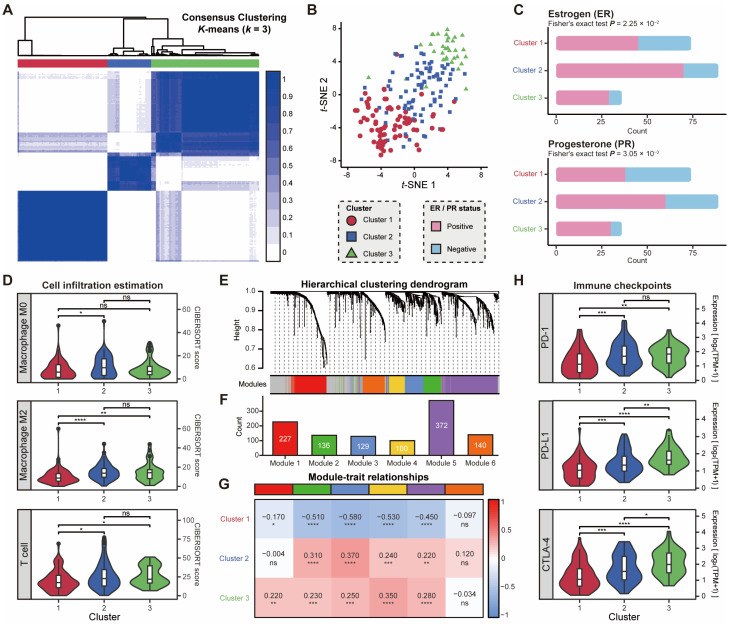
The correlation between lncRNA expression patterns and immune activation in breast cancer. (**A**) A consensus clustering matrix based on lncRNA expression levels, and the clustering method is K-means, where K = 3; (**B**) Distribution of three clusters after the *t*-SNE dimensionality reduction of lncRNA expression, where each point corresponds to one sample; Comparison of (**C**) hormonal characteristics and (**D**) cell infiltration estimation scores in three clusters; (**E**) Cluster dendrogram of hierarchical clustering based on lncRNA expression levels; (**F**) Number of lncRNAs per module in hierarchical clustering; (**G**) Correlation between the six modules of hierarchical clustering and the three clusters of consensus clustering; (**H**) Comparison of the expression levels of immune checkpoint-related genes in the three clusters identified by consensus clustering. ER: estrogen receptor; PR: progesterone receptor. The results were summarised with the following symbols: ‘****’ for *p* ≤ 0.0001, ‘***’ for *p* ≤ 0.001, ‘**’ for *p* ≤ 0.01, ‘*’ for *p* ≤ 0.05 and ’ns’ for *p* > 0.05.

To more sufficiently explore the potential functions and clinical relevance that were not unraveled in the protein-coding genes, breast cancer samples in the SEU-BRCA dataset were clustered based on the expression profiles of both novel and annotated lncRNAs. Using the consensus clustering method, the samples in SEU-BRCA were classified into three clusters with the assurance that the number of samples in each cluster did not deviate significantly (Figure 5A, Methods). Then, the three clusters were also confirmed to be distinguished after dimensionality reduction of the expression matrix of lncRNAs by *t*-SNE (Figure 5B), which further validated the accuracy of the classification. To investigate the differences in biological functions between clusters, we explored the immunohistochemical markers in the different clusters. Our findings revealed that there were significant differences in hormone receptors, such as ER and PR ($p=2.25\times 10^{-2}$ and $p=3.05\times 10^{-3}$, Fisher’s exact test, respectively, Figure 5C), which indicated that the expression pattern of lncRNAs could reflect the hormonal characteristics of breast cancer samples.

Additionally, a total of 23 genes exhibited significantly differential expression patterns between the two clusters ($\left|{log}_{2}FoldChange\right|>1$ and FDR-adjusted p<0.01). Ten of these genes were associated with immune function, with a tendency for expression levels to decrease from cluster 1 to cluster 2, then to cluster 3, while others exhibited no significant trend in expression levels (Supplementary Figure S3). Moreover, after calculating the fractions of immune cells in each sample using CIBERSORT, it was found that three clusters differed in the fractions of M2 macrophages and T cells (Figure 5D). Cluster 1 was observed to have lower fractions of M2 macrophages and T cells compared to the other two clusters, suggesting the potential alteration of the immune mechanisms for each cluster. 

To gain further insight into the distinctions in immune response across the three clusters, we investigated the correlation between the classification outcomes and six co-expression modules constructed with 1509 immune-related genes from the ImmPort database (Figure 5E,F). The results of the functional enrichment analysis (Supplementary Figure S4) confirmed that Module 1 was mainly enriched in the natural killer cell-associated activation pathway and the JAK-STAT cytokine signaling pathway. Module 2 was concentrated on the cell migration, angiogenesis, and epithelial cell proliferation pathway. Module 3 was enriched in the cell differentiation, proliferation, growth, and apoptosis pathway. Module 4 was associated with the Fc receptor signaling pathway, T-cell activation, and cytokine production. Module 5 was linked to humoral immunity-related processes, such as complement activation. Module 6 was related to T-cell activation, leukocyte proliferation, leukocyte adhesion, and interferon response processes. The comparison between the three clusters and the six modules revealed that the intensity of immune activity was weaker in Cluster 1 than in the other two clusters (Figure 5G), confirming the previous discovery that the immune cell fraction was lower in Cluster 1. Meanwhile, Cluster 2 and Cluster 3 principally differed between B-cell-dominated humoral immune-related processes (Module 1) and natural killer cell-dominated non-specific immune processes (Module 5). Furthermore, to probe the potential clinical relevance of the three clusters, we compared 49 immune checkpoint genes [45] across the different clusters and found that 48 immune checkpoint genes exhibited significant differences in expression levels between the two clusters (p<0.01, Wilcoxon signed-rank test, Supplementary Table S1). Specifically, six immune checkpoint genes differed significantly in expression in all clusters (p<0.01, Wilcoxon signed-rank test, Supplementary Figure S5), including *BTN2A1*, *CD47* (known as IAP), *CD80* (known as CTLA-4 Counter-Receptor B7.1), *CD160* (known as BY55), *CD226* (known as DNAM-1), and *CD274* (known as PD-L1). Collectively, the PD-1/PD-L1 and CTLA-4 (Figure 5H), which are commonly employed in cancer immunotherapy, exhibited notable distinctions between the three clusters, suggesting that the expression patterns of lncRNAs were associated with the immune checkpoints in cancer. Therefore, lncRNAs were associated with altered expression of immune checkpoint genes and inhibitory pathways and could potentially mediate the functional state of immunologic cells and its expression patterns could have significant implications in clinical immunotherapy [46].

## 3. Discussion

As an extraordinary category of transcripts, lncRNAs play essential roles in a wide array of cellular processes, which are involved in the regulation of gene expression at various levels, from chromosomal interactions to post-translational modifications [7]. Despite the considerable attention devoted to the potential of annotated lncRNAs as biological markers of breast cancer, there remains a significant gap in our knowledge of the novel lncRNA transcripts that were poorly recorded. Meanwhile, lncRNAs can exhibit significant variability across different datasets [47]. Consequently, a comprehensive analysis of the expression levels and functional annotation of lncRNAs in breast cancer could facilitate further knowledge of breast cancer heterogeneity and provide new insights for their clinical applications in disease risk prediction, disease diagnosis, disease treatment, and prognosis.

In our study, we constructed a systematic pipeline for identifying novel lncRNAs based on RNA-seq data (Figure 1A) and applied it in a breast cancer cohort (SEU-BRCA), which comprised 199 breast cancer samples. A total of 9831 novel lncRNA transcripts from 9108 novel lncRNA genes were identified, which implies that some of these novel lncRNA genes yield multiple variable splicing transcripts. Then, we characterized the novel lncRNA transcripts in four aspects to verify the reliability of the identification pipeline. It was indicated that the identified novel lncRNAs shared similarities with the annotated lncRNAs and exhibited significant differences from the protein-coding transcripts in all four feature statistics above. In particular, in contrast to mRNAs, both novel and annotated lncRNAs exhibited a shorter average length and a markedly lower exon number, indicating that variable splicing could be less abundant in lncRNA gene transcripts [48]. Although the expression levels of novel and annotated lncRNAs were significantly lower than that of mRNA, the differences in their expression across tissues or cell types were considerably greater [49,50]. Thereby, our novel lncRNA identification pipeline was demonstrated to be plausible, and the expression patterns of the novel lncRNAs identified by it in SEU-BRCA were tissue-specific, with potential roles in biological function and regulatory mechanism.

Since the roles of the annotated lncRNAs have been well studied, it was possible to elucidate the regulatory functions of these novel lncRNAs by comparing them with epigenetic features. Of these, antisense and sense overlapping novel lncRNAs accounted for more than the intergenic novel lncRNAs, which was inconsistent with the distribution pattern of the annotated lncRNAs. Particularly, we have discovered that these novel lncRNAs were primarily in introns and physically biased towards TSSs of their host genes. Then, we uncovered that the expression levels of four intronic lncRNAs were significantly correlated with those of their host genes, suggesting that these intronic lncRNAs could potentially impact the transcription levels of host genes. Therefore, we assumed that the difference was attributed to the tissue-specific expression pattern of lncRNAs in breast cancer, and to validate this hypothesis, we performed functional enrichment analysis of the protein-coding genes in which the novel lncRNAs were located. It was evident that several relevant pathways that have been widely discussed in cancer research appeared in the significantly enriched pathways, including the Wnt signaling pathway, Ras protein signal transduction, ErbB signaling pathway, proteoglycans in cancer, and FOXO signaling pathway. Additionally, expression correlation analysis between the intronic lncRNAs and the corresponding protein-coding genes yielded four novel lncRNAs that were significantly and strongly correlated with cancer-related genes, which were discovered to be relevant to carcinogenesis. These findings collectively indicated that the genomic distribution of novel lncRNAs that differed from annotated lncRNAs was not arbitrary, suggesting that these novel lncRNAs could play essential roles in various biological processes of breast cancer.

To further investigate the regulatory mechanisms involved in the identified novel lncRNAs, we intentionally aligned these lncRNAs with known putative enhancers. Of all the novel lncRNAs identified in SEU-BRCA, more than 40% overlapped with breast-cancer-related enhancers at different genomic locations. Additionally, the genes targeted by the novel lncRNAs with putative enhancer features exhibited a bias for enrichment on the X chromosome and these genes with high-frequency presences were significantly associated with the vital pathways related to the proliferation and metastasis of breast cancer, such as vesicle-mediated transport, the VEGFA-VEGFR2 signaling pathway, intrinsic apoptotic signaling pathway, and negative regulation of cell cycle. Previous studies have also confirmed that some of the lncRNAs associated with breast cancer could be associated with the X chromosome; for example, the lncRNA XIST, known for its role in X chromosome inactivation, could participate in regulating breast cancer development through its interaction with BRCA1, influencing cell proliferation, differentiation, and other crucial cellular functions [51]. Moreover, the novel lncRNAs with putative enhancer features were significantly enriched for a motif associated with breast cancer proliferation, and their co-expressed genes were identified to be associated with immune-related responses. Therefore, it was demonstrated that a subset of the novel lncRNAs that overlapped with putative enhancers was potentially involved in the recruitment of transcription factors and the facilitation of chromatin looping between enhancers and promoters to activate gene transcription.

PAM50 is a molecular subtyping criterion that comprises five intrinsic subtypes of breast cancer with different biological characteristics and prognosis [43]. To uncover the elaborate subtype-specific biological functions and regulatory mechanisms of lncRNAs, we characterized the expression patterns of lncRNAs and the associated regulatory networks in individual breast cancer subtypes. After subtype-specific screening of both novel and annotated lncRNAs, we observed that the lncRNAs in subtype Basal-like and subtype pair Luminal A and Luminal B were significantly differentiated from the other subtypes or subtype pairs. The RNA-seq data of primary breast cancer tissue samples from TCGA were used to identify the specifically expressed genes, and the trend in the distribution of subtypes or subtype pairs of specifically expressed genes was found to be in general accordance with the distribution of the genes obtained in SEU-BRCA. Then, we constructed co-expression networks based on the co-expression relationship of these lncRNAs with protein-coding genes and discovered that the regulatory network of subtype Basal-like was primarily connected by negative correlation edges, while that of subtype pair Luminal A and Luminal B was mainly positively connected. The protein-coding genes negatively linked to lncRNAs in the subtype Basal-like were found to play an inhibitory role in the pathways associated with breast cancer initiation and progression, particularly the estrogen-dependent gene expression pathway, which is associated with estrogen response. Furthermore, the protein-coding genes positively linked to lncRNAs in the subtype pair Luminal A and Luminal B could be involved in the phenotype generation of the ER-positive breast cancer tumor cells and play a significant role in hormone therapy resistance.

Our study has demonstrated that lncRNAs exhibited expression differences related to hormone response in PAM50 subtyping criteria. Consequently, we proceeded to perform clustering using lncRNA transcriptional profiles in SEU-BRCA with the objective of detecting subtype-specific regulatory mechanisms of lncRNAs in breast cancer. The breast cancer samples were divided into three clusters using consensus clustering, and the differences between the clusters were validated using t-SNE. Then, the components of the immunohistochemical markers (ER and PR) were revealed to be different for breast cancer samples in each cluster, and 23 genes with significantly different expression levels were found. Some of these genes presented a trend of increased expression from Cluster 1 to Cluster 2 and Cluster 3 and were found to be correlated with the immune response of breast cancer. In addition, the comparison of the immune cell infiltrates between the three clusters also revealed a significantly lower percentage of Cluster 1 compared to Cluster 2 and Cluster 3, suggesting that lncRNA expression patterns could delineate the immune response and progression states of breast cancer [52]. Furthermore, to further explore the immune mechanisms of these three clusters, we calculated the correlation of six co-expression modules constructed using immune-related genes with three clusters. It was demonstrated that Cluster 1 exhibited a lower intensity of immune activity than the other clusters, and the difference between Cluster 2 and Cluster 3 was pertinent to B-cell-dominated humoral immune-related processes and natural killer cell-dominated non-specific immune processes. Comparisons of immune checkpoint genes between three clusters also revealed that the expression levels of almost all genes were significantly distinct, and in particular, six immune checkpoint-related genes differed significantly in all clusters. Specifically, PD-1, PD-L1, and CTLA-4 are commonly employed in cancer immunotherapy [53]. Taken together, our study uncovered that the lncRNAs played an essential role in tumor immunotherapy by regulating immune genes and pathways, and by mediating the functional state of immunologic cells, and their expression patterns could have significant implications in clinical immunotherapy.

Although we performed bioinformatics analyses as comprehensively as possible, further biological experiments are still needed in the future to validate our conclusions. For instance, the analysis of lncRNAs with enhancer features will be further improved by the introduction of public datasets (e.g., GRO-seq) that not only cover RNA-seq. In addition, in the unsupervised clustering analysis based on lncRNA expression patterns, a more intensive elucidation of the specific functional mechanisms of lncRNA genes is required. Ultimately, the study focused exclusively on Chinese breast cancer patients, and additional data from different populations need to be incorporated to enhance the analysis.

## 4. Materials and Methods

### 4.1. Raw Data Processing

In our study, the RNA-seq data of 199 breast cancer patients, named SEU-BRCA, were derived from the National Genomics Data Center (NGDC, https://ngdc.cncb.ac.cn/bioproject/, accessed on 18 August 2021, BioProject number: PRJCA005965, GSA-Human number: HRA001100). For each sample, FastQC (version 0.11.6) was employed to quality control the raw reads, and Trimmomatic (version 0.38) was used to filter the raw sequencing data to remove low-quality, too-short, and unpaired reads, as well as to trim low-quality bases at both ends of the read. Then, reads were aligned to the human reference genome (GRCh37 Ensembl release 75) using HISAT2 (version 2.1.0). Htseq-count (version 0.11.2) and salmon (version 1.3.0) were performed to quantify the genes and transcripts, respectively. StringTie (version 1.3.4d) was used to reconstruct and merge single-sample transcripts to obtain a consensus transcriptome.

### 4.2. Identification of Novel lncRNA Transcripts

The identification of novel lncRNA transcripts was divided into four aspects: (1) The reconstructed transcriptome was compared with annotation files, including the human genome annotation file in GENCODE and the human non-coding gene annotation file in NONCODE, using cuffcompare (version 2.2.1). Transcripts flagged as potential novel transcripts with classification codes ‘i’, ‘j’, ‘x’, ‘o’, and ‘u’ were retained; (2) Transcripts less than 200 nucleotides in length were removed; (3) Transcripts that did not present in homology searches in Pfam and Rfam, and with protein-coding probabilities below 0.1 in CPAT, CPC2, transdecoder, and txCDSpredict, were retained; (4) Single-exon transcripts longer than one thousand nucleotides were removed, as well as transcripts that overlapped with repetitive sequence regions and genomic gap regions from UCSC.

### 4.3. Function Analysis of Novel lncRNAs Associated Protein-Coding Genes

Based on the genomic location of the novel lncRNAs, the associated protein-coding genes that overlapped with them were obtained. Then, clusterProfiler (version 3.14.3) was used to perform KEGG and GO enrichment analysis for the novel lncRNAs.

### 4.4. Extraction of Breast-Cancer-Related Putative Enhancers

The breast-cancer-related putative enhancers were retrieved from ROADMAP and Enhancer Atlas 2.0. The three mammary tissue or cell types, containing E027, E028, and E119, as well as immune cells [54], including E029, E030, E032, E034, E037, E038, E039, E040, E043, E044, E045, E046, E047, E048, and E062, were obtained from ROADMAP. Putative enhancers were screened based on chromatin state (such as 6 genic enhancers, 7 enhancers, and 12 bivalent enhancers) in the core 15-state model. Additionally, the rest of the breast-cancer-related putative enhancers were obtained from cell lines, including MCF-10A, MCF-7, ZR-75-1, HCC-1954, MDA-MB-231, T47D, and ZR-75-30, and immune cell CD4+ of Enhancer Atlas 2.0.

The target genes of enhancers that overlapped with novel lncRNAs were obtained from Enhancer Atlas 2.0 and the enhancer–target interaction pairs with scores greater than 1 were retained. Genes with a frequency greater than 5 were selected for downstream analysis.

In the expression correlation calculations between novel lncRNAs with positional association with putative enhancer regions and target genes regulated by their corresponding putative enhancers, the novel lncRNA-gene pairs with absolute Pearson correlation coefficients greater than 0.5 were retained.

### 4.5. Detection of Subtype-Specific Expressed lncRNAs

To yield the PAM50-based subtyping results for 199 breast cancer samples, genefu (version 2.18.1) was used. Given the low expression of lncRNAs and the possibility that their downregulation could be attributed to sequencing inaccuracy, which could result in an inaccurate reflection of the differences across subtypes, the study focused on the significantly upregulated lncRNAs. Then, limma (version 3.42.2) was utilized to identify differently expressed lncRNAs across subtypes. Firstly, differential expression analysis was performed for individual subtypes (and subtype pairs) against the other subtypes, where the Fold Change was greater than 5 and the FDR-adjusted *p* was less than 0.01. Next, the Kruskal–Wallis test was applied to ensure no differences among the rest of the subtypes (Supplementary Table S2), where *p* was greater than 0.05. This approach could guarantee that these genes were specifically highly expressed only in the assigned subtype or subtype pair with no differences across the rest subtypes.

### 4.6. Construction of lncRNA Regulatory Networks

Initially, protein-coding genes associated with the lncRNAs that exhibited specific expression in individual subtypes or subtype pairs were extracted, where the absolute Pearson correlation coefficient was greater than 0.5 and *p* was less than 0.05.

### 4.7. Consensus Clustering Based on Expression Patterns of lncRNAs

In order to accurately construct the lncRNA expression matrix of 199 breast cancer samples, lncRNAs with expression values of zero were excluded, and then the top 5000 lncRNAs were chosen for consensus clustering based on the median absolute deviation of lncRNA expression in each sample. Based on the principle that there was no significant imbalance in the number of samples within each cluster, K = 3, which was greater than 2, and the cluster consistency of each cluster was higher than 0.8, was taken as the optimal cluster count for consensus clustering. In addition, CIBERSORT was applied to calculate the fractions of 22 immune cells in each sample, and the differences between the three clusters were compared.

### 4.8. Construction of Co-Expression Module of Immune-Related Genes

To explore the differences in the immunological mechanisms across the three clusters, the 1509 immune-related genes were obtained from ImmPort and the expression matrix of these genes was extracted from SEU-BRCA. Then, six co-expression modules were constructed using WGCNA (version 1.69), and the expression correlations between the modules and clusters were calculated.

### 4.9. Statistical Analysis

Continuous variables were compared by using the Chi-square test, Wilcoxon signed-rank test, Kruskal–Wallis test, and Pearson correlation coefficient, and categorical variables were compared using the Fisher’s exact test. The statistical significance threshold was set at *p* < 0.05. The false discovery rate (FDR) procedure was used in multiple hypothesis testing to reduce false positive rates. Statistical analyses were performed with R (version 3.6.1).

## 5. Conclusions

In conclusion, our study constructed a novel lncRNA identification pipeline and applied it to a breast cancer cohort (SEU-BRCA), yielding 9831 novel lncRNA transcripts from 9108 novel lncRNAs. Then, we explored the genomic and chromatin features of the identified lncRNAs, as well as the regulatory mechanisms of the lncRNAs with putative enhancer features, which could promote enhancer–promoter looping and transcriptional activation of target genes. Moreover, we uncovered that specifically expressed lncRNAs in subtypes or subtype pairs were responsible for the inhibition or maintenance of ER- and PR-positive phenotypes of breast cancer and associated with hormone response. Finally, we revealed that the expression pattern of lncRNAs could elucidate the immune process of breast cancer. Consequently, lncRNAs could be utilized as potential biomarkers or immunotherapeutic targets for the diagnosis and prognosis of breast cancer.

## Data Availability

The RNA-seq data of 199 breast cancer patients are available from the National Genomics Data Center (NGDC, https://ngdc.cncb.ac.cn/bioproject/, accessed on 18 August 2021) with BioProject Accession: PRJCA005965 and GSA for Human Accession: HRA001100.

## Supplementary Materials

The supporting information can be downloaded at: https://www.mdpi.com/article/10.3390/ijms25158456/s1.
