# Supplementary material for: Comprehensive Transcriptome Analysis Expands lncRNA Functional Profiles in Breast Cancer
Source: Int J Mol Sci. 2024 Aug 2;25(15):8456. doi: 10.3390/ijms25158456 (PMC11313538; doi:10.3390/ijms25158456)
Supplement: Supplementary file 1 [file ijms-25-08456-s001.zip › Supplementary Figure S1-S5.pdf]

c

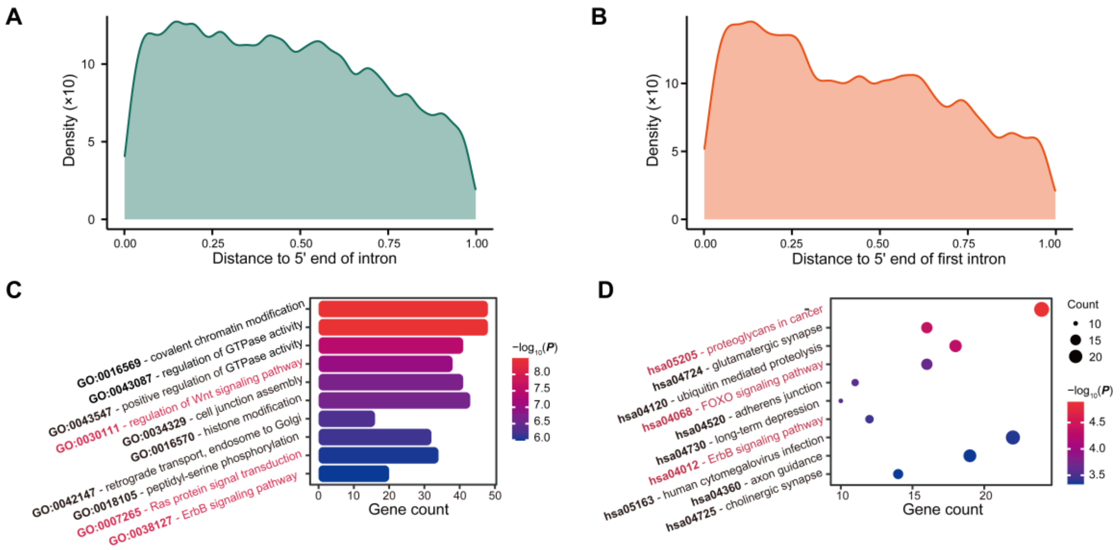

Supplementary Figure S1. Normalized distance from the middle points of intronic lncRNAs located in the (A) introns, or (B) first introns to the 5' end of the corresponding host genes; (C) GO enrichment analysis and (D) KEGG pathway enrichment analysis of protein-coding genes located by intronic lncRNAs, where the pathways marked in red are frequent in cancer research.

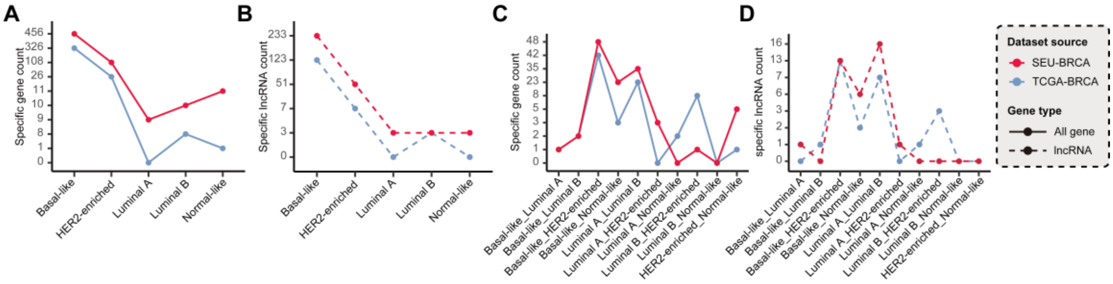

Supplementary Figure S2. Comparison of the number of specifically expressed genes and lncRNA genes of different (A-B) subtypes and (C-D) subtype pairs in SEU-BRCA and TCGA-BRCA. The red lines represent SEU-BRCA, and the blue lines denote TCGA-BRCA. Solid lines refer to the number of all genes and dashed lines refer to the number of lncRNAs. The differential expression analysis tool used for the above pre-experiments was DESeq2.

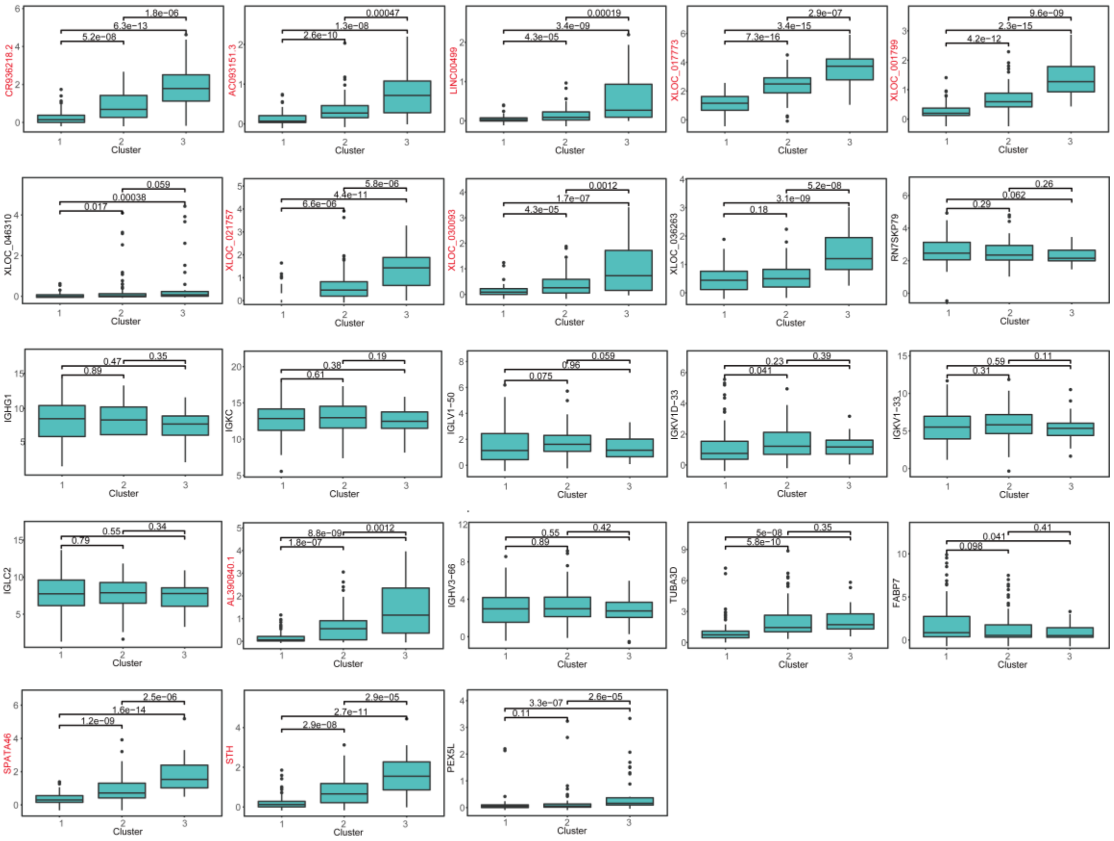

Supplementary Figure S3. Comparison of expression levels of 23 differentially expressed genes among clusters. Differentially expressed genes obtained after two-by-two comparative analysis of three clusters, including CR936218.2, AC093151.3, LINC00499, XLOC_017773, XLOC_001799, XLOC_046310, XLOC_021757, XLOC_030093, XLOC_036263, RN7SKP79, IGHG1, IGKC, IGLV1-50, IGKV1D-33, IGKV1-33, IGLC2, AL390840.1, IGHV3-66, TUBA3D, FABP7, SPATA46, STH and PEX5L.

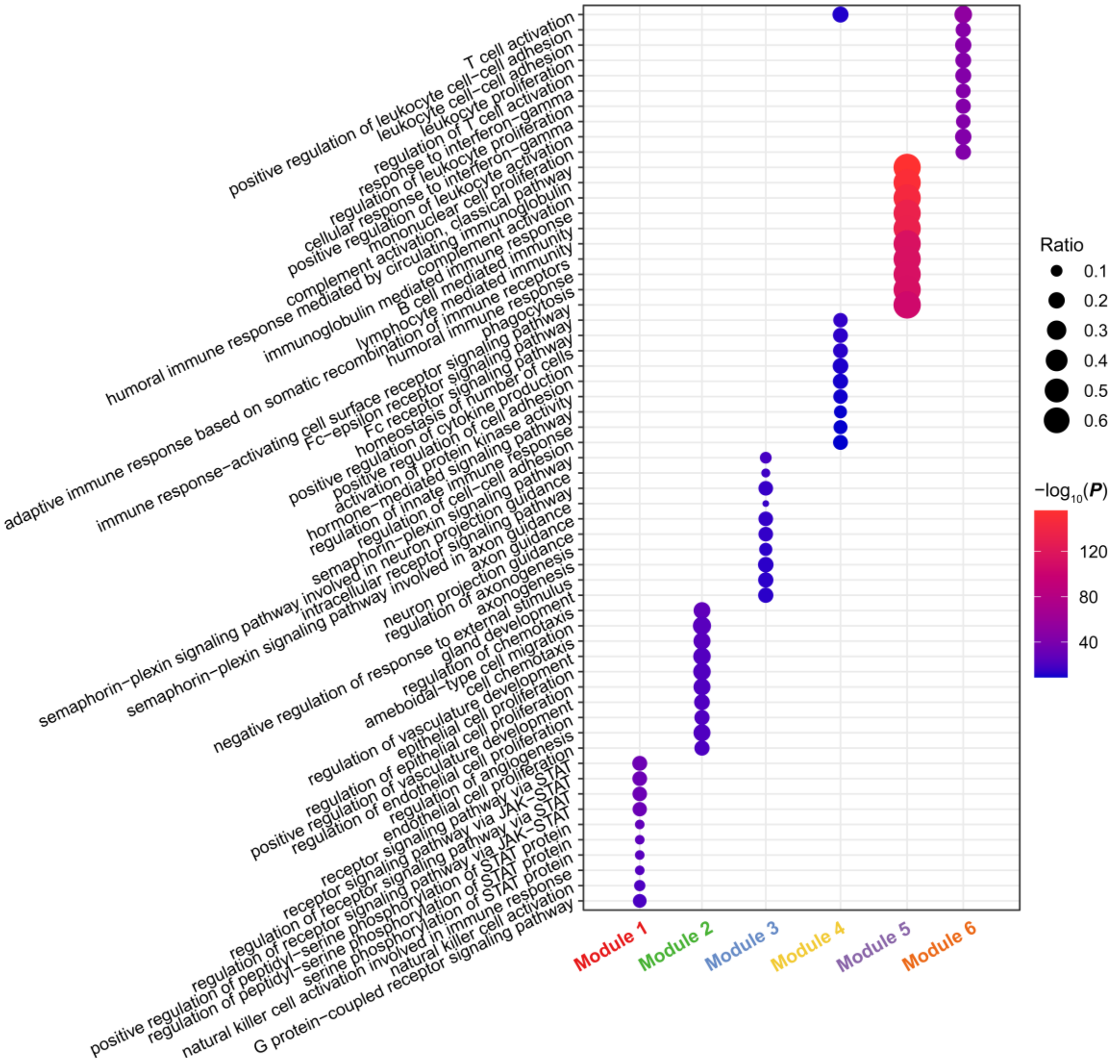

Supplementary Figure S4. Functional enrichment of co-expression modules of immune-related genes.

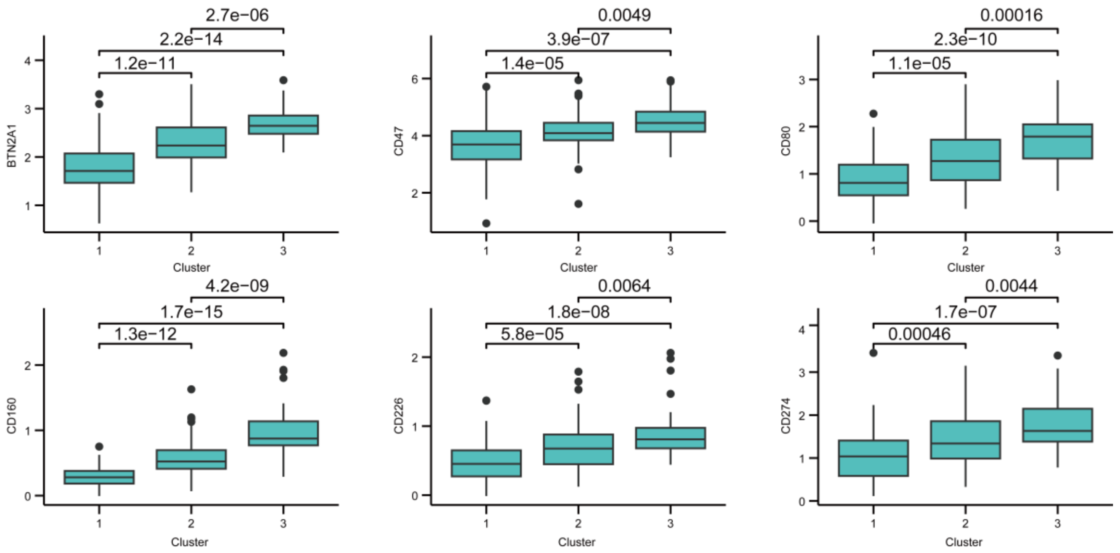

Supplementary Figure S5. Comparison of expression levels of six immune checkpoint genes that were significantly differentially expressed in three clusters.
